# Prevalence and predictors of elective and emergency caesarean delivery among reproductive-aged women in Bangladesh: evidence from demographic and health survey, 2017–18

**DOI:** 10.1186/s12884-022-04833-6

**Published:** 2022-06-24

**Authors:** T. Muhammad, Shobhit Srivastava, Pradeep Kumar, Rashmi Rashmi

**Affiliations:** 1 Department of Family & Generations, International Institute for Population Sciences, Mumbai, India; 2 Department of Survey Research & Data Analytics, International Institute for Population Sciences, Mumbai, India; 3 Department of Population and Development, International Institute for Population Sciences, Deonar East, Mumbai, 400088 Maharashtra India

**Keywords:** C-section delivery, Elective c-section, Emergency c-section, Predictors, Bangladesh

## Abstract

**Background:**

Over the years, an increasing trend of unnecessary caesarean section (c-section) deliveries has raised concerns in Bangladesh. So far, many studies have reported the risk factors of c-section delivery in Bangladesh. However, most of these studies did not estimate the predictors of the two c-section procedures (i.e., emergency and elective) separately based on the timing of the c-section decision. This study solely brings forward the role of socio-demographic and economic factors that may be associated differently with emergency and elective c-section deliveries.

**Methods:**

Data for the study were drawn from the 2017–18 Bangladesh Demographic and Health Survey with 5,299 women aged 15–49 years who gave birth at a health facility during three years preceding the survey. Descriptive statistics along with bivariate analysis were used to fulfill the study objectives. Further, multivariable logistic regression analysis was conducted on binary outcome variables of elective/emergency c-section deliveries.

**Results:**

Approximately one-third of women in the reproductive-age group opted for delivery through c-section. Out of them, 18.7% of women had elective c-sections, and 14.1% had emergency c-sections. Women who had mass media exposure were 32% more likely to deliver through elective c-sections than women who had no exposure [AOR: 1.32; CI: 1.02–1.72]. Women with higher education had a 56% lower likelihood of delivering through emergency c-section than women with no educational status [AOR: 0.44; CI: 0.24–0.83]. Children from the third or higher birth order were significantly more likely to be delivered through elective c-sections than those from the first birth order [AOR: 2.67; CI: 1.75–4.05]. In contrast, children with higher birth order had fewer chances of emergency c-section than children with first birth order [AOR: 0.29; CI: 0.18 -0.45]. Both elective and emergency c-section deliveries were significantly higher among private health facilities.

**Conclusion:**

Although c-section delivery has emerged as a life-saving intervention, the overuse of such practice has created lucrative risks for the mother and unborn child. Proper sensitization of mothers and families can enhance the knowledge of the unsafe nature of unnecessary c-section deliveries. Authorizations in case of over-use of elective and emergency c-sections should be observed to minimize the unnecessary c-sections and related complications and to increase normal institutional deliveries in Bangladesh.

## Background

During pregnancy, women face different complications, and in such situations, caesarean section (c-section) delivery is considered the best technique to save the life of mother and child [1, 2]. Globally, an increase in c-section delivery has been observed due to the perception of the safety of healthcare providers and patients, medico-legal concerns, and maternal risks, including obesity, advanced age, and poor medical conditions [3–5]. According to the World Health Organization (WHO) report, the acceptable c-section delivery rate is 10–15%, and more than 15% rates are considered medically unjustified [6]. On the other hand, the c-section delivery rate in Bangladesh increased from 17.7% in 2012 to 35.4% in 2019 [7].

The urgency or timing of a caesarean section can be classified into elective and emergency c-section procedures. Elective or planned caesarean section is a surgery planned in advance before labor onset. On the other hand, an emergency caesarean section is a surgery undertaken before or in labor due to the immediate concerns for the mother or the fetus [8]. A review of 17 studies suggests that several personal and societal reasons, including fear of pain during childbirth and perceived inequality and inadequacy of care, contributed to the increased incidence of c-section delivery on maternal requests [9]. Similarly, women who had a previous c-section delivery made the largest contribution to the overall c-section delivery rate [10–12].

Furthermore, the decision on elective c-section delivery is influenced by some cultural factors, including religious acceptance and societal attitudes towards such procedures, previous experiences, and interactions with health care professionals [13]. The reasons for such elective c-sections may also include pelvic floor protection, convenience, and the reduced risk of some fetal injuries [14, 15]. Studies have examined the association between advanced maternal age and adverse pregnancy outcomes and elective or emergency c-section delivery and found an increase in c-section deliveries [16, 17]. The study reveals the relative safety of elective c-section delivery compared to emergency c-section delivery. The perceived advantages led to the widespread acceptance of elective c-section delivery [18].

There are other reasons for the increase in the c-section delivery rate that include higher socioeconomic status and cultural and social environments [2, 19]. Studies in developed and developing countries found that women from higher socioeconomic backgrounds delay childbearing until they reach the age of 30 years or older to complete their education, secure a job, and become financially stable before having children [20–22]. And such delays in pregnancy may lead to complications for the child and mother; however, modern techniques have brought different solutions for such difficulties, among which elective c-section is also popular. Several studies also found that c-section delivery rates were high among urban and better-off populations than their rural and worse-off counterparts and those who preferred private health care facilities to public facilities [23, 24]. In a cross-sectional study, factors like a mother being older, obese, higher number of antenatal care (ANC) visits, and delivery in a private facility were associated with higher rates of c-section delivery [25]. The influence of partners also plays an important role in the mode of delivery [26].

Although the potential risks of maternal and neonatal morbidities between c-section and vaginal deliveries are still being debated [15], past studies have shown that c-section deliveries, even elective, carry a higher risk to maternal health than vaginal delivery [27–29]. A recent study conducted in Bangladesh found that c-section delivery increased the risk of various childhood diseases by 5 percent [7]. Due to health care providers’ inducements and their financial benefits, there is an increasing trend of c-section deliveries in Bangladesh. The rate is higher than in many other Asian countries [30]. Understanding the specific childbearing characteristics of women of reproductive age and the underlying causes of the increased c-section delivery rates would facilitate targeting the modifiable risk factors in maternal and child health. This would reduce the need for a c-section mode of delivery, thus improving the health of mothers and infants and reducing the length and cost of their hospital admissions.

Thus, this study aims to examine the prevalence of c-section delivery in women in Bangladesh from 2017 to 2018, with additional analyses of the associations of factors such as maternal socio-demographics, health and pregnancy behaviors, physical facilities, and partners’ characteristics on overall c-section delivery and elective and emergency c-section deliveries in particular.

## Data and methods

### Data

Data for this study have been extracted from the 2017–18 Bangladesh Demographic and Health Survey (BDHS), a nationally representative survey of 20,127 women aged 15–49 years from 19,457 households covering 672 sample points (clusters) from both urban and rural areas throughout Bangladesh. The survey was conducted by the National Institute for Population Research and Training (NIPORT) of the Ministry of Health and Family Welfare [31]. The 2017–18 survey used a two-stage stratified sample of households. The survey used the Bangladesh Census as a sampling frame from the list of enumeration areas (EAs) of the 2011 Population and Housing Census of the People’s Republic of Bangladesh, provided by the Bangladesh Bureau of Statistics (BBS). The primary sampling unit (PSU) of the survey is an EA with an average of about 120 households. In the first stage, 675 EAs (250 in urban areas and 425 in rural areas) were selected with probability proportional to EA size. In the second stage of sampling, a systematic sample of an average of 30 households per EA was selected to provide statistically reliable estimates of key demographic and health variables for the country as a whole, for urban and rural areas separately [31]. The BDHS obtained detailed information on fertility levels, marriage, fertility preferences, awareness and use of family planning methods, breast-feeding practices, nutritional status of women and young children, childhood mortality, maternal and child health, and knowledge and attitudes regarding HIV/AIDS and other sexually transmitted infections. The detailed information on the survey are given elsewhere [31]. The effective sample size for the analysis was 5,299 women aged 15–49 years who had given birth at a health facility for three years preceding the survey.

### Variable description

#### Outcome variable

The response variable for this study was c-section deliveries among women age 15–49 years. The respondents were women who had given birth during three years preceding the survey. The question was asked, “was the baby delivered by caesarean section; that is, did they cut your belly open to take the baby out?” The response was 0 “no” and 1 “yes”. If yes, they were asked “when was the decision made for you to have a caesarean section? Was it before the onset of labor or after the onset of labor?” The response was “Before the onset of labor” and “After the onset of labor”. In this study, before the onset of labor refers to ‘elective’ and after the onset of labor refers to ‘emergency’.

#### Explanatory variable

Following are the explanatory variables used in the present study according to the prior literature [1, 24, 32].

1. Respondent’s age (15–19, 20–24, 25–29, and 30–49 years),
2. ﻿Respondent’s educational level (no education, primary, secondary, and higher),
3. ﻿Respondent’s work status (not working and working),
4. ﻿Respondent’s mass media exposure (no exposure and exposure). Questions were asked regarding how often they read newspapers, listened to the radio, and watched television; responses on the frequencies were: almost every day, at least once a week, less than once a week, or not at all; women were considered to have any exposure to mass media if they had exposure to any of these sources and as having no exposure if they responded with ‘not at all’ for all three sources of media,
5. Body mass index (BMI) (underweight, normal, and overweight/obese). Height and weight data were used to calculate BMI, a composite measure of adult nutritional status. Calculations of BMI excluded women for whom there was no information on height and/or weight. Women who were pregnant on the day of the survey visit or had given birth during the preceding 2 months were also excluded. The study considered women underweight if her BMI was less than 18.5 kg/m2, normal if BMI was 18.5–24.9 kg/m2, and overweight or obese if it was more than or equal to 25 kg/m2 [33],
6. Partner’s education (no education, primary, secondary, and higher),
7. Partner’s occupation (unemployed, professional/technical/managerial, sales, agricultural, services, and skilled and unskilled),
8. ﻿Respondent’s age at first birth (less than 18, 18–20, 21–24, and 25 years or more),
9. Birth order (first, second and three or more),
10. ANC visits (no visit, 1–3 visit, and 4 or more),
11. Multiple births (single and multiple),
12. Place of delivery (public and private). Public includes medical college hospital, specialized government hospital, district hospital, maternal and child welfare center, upazilla health complex, other public sector, urban health and family welfare center, community clinic,
13. And private includes private medical college hospital, private hospital, private clinic, other private medical sector, NGO static clinic, delivery hut [31],
14. Wealth index (poorest, poorer, middle, richer, and richest). Households were given scores based on the number and kinds of consumer goods they own, ranging from a television to a bicycle or car, and housing characteristics such as source of drinking water, toilet facilities, and flooring materials. These scores are derived using principal component analysis. National wealth quintiles were compiled by assigning the household score to each usual (de jure) household member, ranking each person in the household population by her or his score, and then dividing the distribution into five equal categories, each comprising 20% of the population [31],
15. Place of residence (urban and rural), and
16. Division (Barisal, Chittagong, Dhaka, Khulna, Mymensingh, Rajshahi, Rangpur, and Sylhet).

### Statistical analysis

Descriptive statistics along with bivariate analysis were used to find out the results. In bivariate analysis, chi-square tests [34] were conducted to examine the association between women’s background characteristics and their c-section deliveries. Multivariable logistic regression [34] analysis was conducted with elective/emergency c-section deliveries as binary outcome variables. The adjusted odds ratio (AOR) was presented with a 95% confidence interval (CI). The model is usually put into a more compact form as follows:

$$\mathrm{ln}\left(\frac{{P}_{i}}{1-{P}_{i}}\right)={\beta }_{0}+{\beta }_{1}{x}_{1}+\dots +{\beta }_{M}{x}_{m},$$

where, ${\beta }_{0},\dots ..,{\beta }_{M}$ are regression coefficients indicating the relative effect of a particular explanatory variable on the outcome variable. These coefficients change as per the context in the analysis in the study. Variance inflation factor (VIF) [34] was estimated to check the multicollinearity and it was found that there was no evidence of multicollinearity in the variable used. Additionally, svyset command in Stata 14 [35] was used to control for complex survey design. Moreover, individual weights were used to generate nationally representative estimates.

## Results

**Fig. 1:**
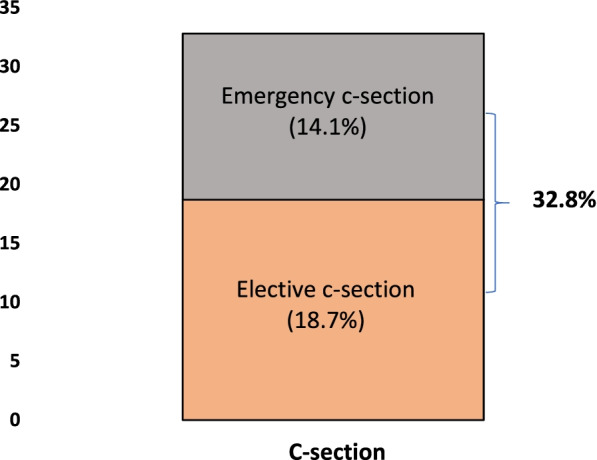
Prevalence of C-section, elective, and emergency c-section in Bangladesh, 2017–18

Figure 1 revealed that 32.8% of women in the reproductive-age group opted for delivery through c-section. Out of 32.8%, about 18.7% of women had elective c-sections, and 14.1% had emergency c-sections.

Table 1 presents the socio-demographic profile of the study population in Bangladesh, 2017–18. About one-fifth of the women were 30–49 years old. Nearly 6.6% of women were not educated and 16.9% of women had a higher educational level. About 36.9% of women were in the working category. Almost 34.8% of women had no exposure to mass media. Nearly, 17.7% of women were underweight, whereas 22.1% of women were either overweight or obese. About 15% of women’s partners were uneducated, and 2.2% of men were unemployed. About 4 in 10 women had age at first birth below 18 years, and the almost same proportion of children was of the first order. About 47% of women had four or more ANC visits. Nearly 2% of women had multiple births. About 84.7% had delivered in the private health facility.

**Table 1:** Socio-demographic profile of the study population, Bangladesh, 2017–18

Background characteristics	Sample	Percentage
**Women’s age (in years)**		
15–19	920	17.9
20–24	1,885	35.2
25–29	1,379	25.8
30–49	1,115	21.0
**Women’s educational level**		
Not educated	346	6.6
Primary	1,477	27.6
Secondary	2,534	48.9
Higher	942	16.9
**Women’s work status**		
Not working	3,337	63.1
Working	1,962	36.9
**Women’s mass media exposure**		
No Exposure	1,921	34.8
Exposure	3,378	65.2
**Women’s Body Mass Index**^a^		
Underweight	821	17.7
Normal	2,689	60.2
Overweight or Obese	995	22.1
**Partner’s education**		
No Education	804	15.0
Primary	1,769	33.5
Secondary	1,722	33.4
Higher	1,004	18.1
**Partner’s occupation**		
Unemployed	122	2.2
Professional/Technical/Managerial	479	8.1
Sales	994	18.2
Agricultural	976	18.9
Services	680	13.3
Skilled And Unskilled	2,048	39.3
**Women’s age at first birth (in years)**		
< 18	2,152	41.7
18–20	2,004	38.0
21–24	841	15.1
≥ 25	302	5.1
**Birth order**		
First	2,065	39.0
2nd order	1,695	32.1
3 or more	1,539	28.9
**ANC visits**^b^		
No visit	408	8.0
1–3 visits	2,188	45.0
4 or more	2,411	47.0
**Multiple births**		
Single	5,190	98.0
Multiple	109	2.0
**Place of delivery**^c^		
Public	803	15.4
Private	1,845	84.7
**Wealth quintile**		
Poorest	1,148	20.8
Poorer	1,089	20.8
Middle	954	19.1
Richer	1,040	20.1
Richest	1,068	19.3
**Place of residence**		
Urban	1,814	26.7
Rural	3,485	73.3
**Division**		
Barisal	561	5.7
Chittagong	886	21.3
Dhaka	778	25.5
Khulna	545	9.0
Mymensingh	633	8.5
Rajshahi	554	11.7
Rangpur	580	10.4
Sylhet	762	8.0
**Total**	5,299	100.0

^a^Exclude currently pregnant and women with a birth in the last 2 months; underweight: < 18.5 kg/ m2, normal: 18.5–24.9 kg/m2, overweight and obese: ≥ 25 kg/m2

^b^last birth sample only

^c^facility delivery; *ANC* Ante-natal care

Table 2 presents the prevalence of c-section deliveries and estimates from multivariable logistic regression analysis by background characteristics in Bangladesh. Women’s age and educational status had no significant association with c-section deliveries. Women who were working had a lower likelihood of delivering through c-section than those not working [AOR: 0.58; CI: 0.51–0.65]. Women who were overweight or obese had a 40% higher likelihood of delivering through c-section than women who had normal BMI [AOR: 1.40; CI: 1.08–1.80]. Husband education and occupation had no significant association with women’s c-section delivery. Women aged 25 years and above, during their first childbirth, had 2.17 times higher odds of c-section delivery than women with age at first birth below 18 years [AOR: 2.17; CI: 1.19 -3.95]. Women with four or more ANC visits had 2.47 times higher odds of c-section delivery than women with no ANC visits [AOR: 2.47; 1.15–5.28]. Women who delivered at a private facility had 6.74 times higher odds of delivering through c-section than women who delivered at a public facility. Women from the richest wealth quintile had higher odds of delivering through c-section than women from the poorest wealth quintile [AOR: 1.89; CI: 1.18–3.03]. Women belonging to the regions of Chittagong [AOR: 0.57; CI: 0.37 -0.87] and Rangpur [AOR: 0.61; CI: 0.38–0.96] had significantly lower odds of c-section delivery than women from Barisal.

**Table 2:** Prevalence of c-section deliveries and estimates from multivariable logistic regression analysis by background characteristics in Bangladesh, 2017–18

Background characteristics	C-Section	AOR [95% CI]
**Women’s age (in years)**		
15–19	30.5	Ref
20–24	34.5	1.16(0.82 -1.64)
25–29	31.4	0.96(0.6 -1.53)
30–49	33.6	1.28(0.73 -2.25)
**Women’s educational level**		
Not educated	16.4	
Primary	17.9	Ref
Secondary	34.1	0.64(0.35 -1.18)
Higher	59.7	0.66(0.34 -1.29)
**Women’s work status**		
Not working	37.3	Ref
Working	25.1	0.58***(0.51 -0.65)
**Women’s mass media exposure**		
No Exposure	17.0	Ref
Exposure	41.2	1.26(0.97 -1.64)
**Women’s Body Mass Index**		
Underweight	25.7	0.98(0.73 -1.31)
Normal	29.2	Ref
Overweight or Obese	50.3	1.40**(1.08 -1.80)
**Partner’s education**		
No Education	18.2	Ref
Primary	22.1	0.73(0.49 -1.1)
Secondary	35.4	0.78(0.52 -1.18)
Higher	60.0	0.96(0.59 -1.55)
**Partner’s occupation**		
Unemployed	32.0	Ref
Professional/Technical/Managerial	62.3	1.20(0.56 -2.59)
Sales	37.7	1.13(0.55 -2.31)
Agricultural	20.8	1.27(0.61 -2.63)
Services	34.9	1.51(0.73 -3.12)
Skilled And Unskilled	29.5	1.12(0.56 -2.24)
**Women’s age at first birth (in years)**		
< 18	24.8	Ref
18–20	31.8	1.04(0.8 -1.37)
21–24	45.5	1.37(0.94 -1.98)
> 25	67.5	2.17**(1.19 -3.95)
**Birth order**		
First	40.3	Ref
2nd order	34.4	1.20(0.87 -1.64)
3 or more	20.8	0.87(0.55 -1.38)
**ANC visits**		
No visit	6.0	Ref
1–3 visits	23.8	1.72(0.81 -3.67)
4 or more	46.9	2.47**(1.15 -5.28)
**Multiple births**		
Single	32.4	Ref
Multiple	50.1	1.41(0.62 -3.19)
**Place of delivery**		
Public	35.2	Ref
Private	78.6	6.74***(5.42 -8.38)
**Wealth quintile**		
Poorest	13.0	Ref
Poorer	22.3	1.28(0.87 -1.9)
Middle	30.9	1.29(0.86 -1.93)
Richer	38.2	1.12(0.74 -1.69)
Richest	61.5	1.89***(1.18 -3.03)
**Place of residence**		
Urban	43.8	0.83(0.65 -1.06)
Rural	28.7	Ref
**Division**		
Barisal	24.5	Ref
Chittagong	26.2	0.57**(0.37 -0.87)
Dhaka	42.7	1.13(0.72 -1.76)
Khulna	42.7	1.22(0.78 -1.92)
Mymensingh	26.1	1.17(0.72 -1.88)
Rajshahi	35.6	1.00(0.63 -1.58)
Rangpur	27.8	0.61**(0.38 -0.96)
Sylhet	22.6	1.10(0.68 -1.78)

*C-section* Caesarean Section; *p*-values based on chi-square test; *AOR* Adjusted Odds Ratio, *CI* Confidence Interval; ***p < 0.001; **p < 0.05; *ANC *Ante-natal care, *Ref* Reference; the logistic regression estimates are controlled for all the background characteristics

Table 3 reveals the prevalence of elective and emergency c-section deliveries and estimates from multivariable logistic regression analysis by background characteristics in Bangladesh. It was found that women’s age does not have any significant association with elective or emergency c-section deliveries. Women’s educational status had no significant association with elective c-section deliveries. However, women with higher educational status had a 56% significantly lower likelihood of delivering through emergency c-section than women with no educational status [AOR: 0.44; CI: 0.24–0.83]. Women who had mass media exposure were 32% more likely to deliver through elective c-section than women who had no exposure [AOR: 1.32; CI: 1.02–1.72]. Partner’s education and occupational status had no significant effect on women delivering through elective or emergency c-sections.

**Table 3:** Prevalence of elective and emergency c-section deliveries and estimates from multivariable logistic regression analysis by background characteristics in Bangladesh, 2017–18

Background characteristics	Elective c-section		Emergency c-section	
	**%**	**AOR [95% CI]**	**%**	**AOR [95% CI]**
**Women’s age (in years)**				
15–19	12.1	Ref	18.4	Ref
20–24	19.4	1.23(0.87 -1.73)	15.1	1.04(0.74 -1.44)
25–29	19.3	0.83(0.53 -1.28)	12.2	1.43(0.91 -2.23)
30–49	22.4	0.89(0.53 -1.50)	11.1	1.65(0.95 -2.86)
**Women’s educational level**				
Not educated	7.8	Ref	8.7	Ref
Primary	9.1	0.86(0.48 -1.56)	8.8	0.71(0.40 -1.27)
Secondary	18.5	1.03(0.58 -1.83)	15.6	0.63(0.36 -1.11)
Higher	39.1	1.44(0.78 -2.69)	20.6	0.44**(0.24 -0.83)
**Women’s work status**				
Not working	21.5	Ref	15.8	Ref
Working	13.8	0.59***(0.50 -0.68)	11.2	0.70**(0.59 -0.83)
**Women’s Mass media exposure**				
No Exposure	7.5	Ref	9.6	Ref
Exposure	24.7	1.32**(1.02 -1.72)	16.5	0.94(0.72 -1.22)
**Women’s Body Mass Index**				
Underweight	13.0	1.06(0.79 -1.41)	12.7	0.92(0.69 -1.24)
Normal	16.4	Ref	12.8	Ref
Overweight or Obese	31.8	1.21(0.97 -1.51)	18.5	1.05(0.83 -1.33)
**Partner’s education**				
No Education	8.1	Ref	10.1	Ref
Primary	10.9	0.99(0.66 -1.48)	11.2	0.75(0.5 -1.11)
Secondary	19.7	1.10(0.73 -1.67)	15.6	0.72(0.48 -1.07)
Higher	40.0	1.46(0.92 -2.32)	20.0	0.60(0.38 -1.05)
**Partner’s occupation**				
Unemployed	18.5	Ref	13.5	Ref
Professional/Technical/Managerial	39.7	0.89(0.43 -1.86)	22.6	1.38(0.65 -2.93)
Sales	24.0	1.39(0.69 -2.81)	13.7	0.81(0.39 -1.66)
Agricultural	10.9	1.20(0.58 -2.47)	9.9	1.09(0.53 -2.25)
Services	17.9	1.16(0.57 -2.38)	17.0	1.34(0.65 -2.75)
Skilled And Unskilled	15.9	1.01(0.51 -2.01)	13.6	1.17(0.59 -2.35)
**Women’s age at first birth (in years)**				
< 18	12.6	Ref	12.2	Ref
18–20	18.1	1.14(0.88 -1.47)	13.7	0.90(0.69 -1.18)
21–24	27.6	1.58***(1.14 -2.2)	17.9	0.77(0.54 -1.1)
> 25	46.1	2.75***(1.67 -4.55)	21.4	0.53**(0.31 -0.92)
**Birth order**				
First	18.8	Ref	21.5	Ref
2nd order	23.2	2.58***(1.94 -3.43)	11.2	0.39***(0.29 -0.54)
3 or more	13.5	2.67***(1.75 -4.05)	7.3	0.29***(0.18 -0.45)
**ANC visits**				
No visit	3.4	Ref	2.6	Ref
1–3 visits	12.8	1.23(0.54 -2.81)	10.9	1.57(0.65 -3.77)
4 or more	27.3	1.51(0.66 -3.47)	19.6	1.78(0.74 -4.3)
**Multiple births**				
Single	18.2	Ref	14.2	Ref
Multiple	40.9	1.55(0.74 -3.25)	9.2	0.84(0.35 -1.99)
**Place of delivery**				
Public	18.9	Ref	16.3	Ref
Private	45.3	2.91***(2.31 -3.65)	33.3	3.23***(2.5 -4.17)
**Wealth quintile**				
Poorest	6.0	Ref	7.0	Ref
Poorer	10.6	1.12(0.74 -1.7)	11.7	1.22(0.82 -1.81)
Middle	14.7	1.05(0.69 -1.59)	16.2	1.33(0.89 -2)
Richer	22.9	1.22(0.8 -1.85)	15.3	0.99(0.65 -1.51)
Richest	40.5	1.64**(1.04 -2.6)	21.0	1.09(0.68 -1.73)
**Place of residence**				
Urban	27.0	0.92(0.74 -1.15)	16.9	0.90(0.71 -1.13)
Rural	15.7	Ref	13.1	Ref
**Division**				
Barisal	14.3	Ref	10.2	Ref
Chittagong	15.1	0.62**(0.41 -0.93)	11.0	0.98(0.63 -1.52)
Dhaka	25.6	0.95(0.64 -1.42)	17.1	1.17(0.76 -1.81)
Khulna	23.7	0.91(0.61 -1.38)	19.0	1.32(0.85 -2.04)
Mymensingh	13.7	0.91(0.58 -1.4)	12.3	1.31(0.83 -2.09)
Rajshahi	18.7	0.81(0.53 -1.23)	16.9	1.29(0.83 -2.02)
Rangpur	15.7	0.67(0.44 -1.04)	12.2	0.94(0.59 -1.5)
Sylhet	12.6	0.86(0.55 -1.34)	10.0	1.27(0.79 -2.04)

*C-section* Caesarean section, *AOR* Adjusted Odds Ratio, *CI* Confidence Interval; ***p < 0.001; **p < 0.05; *ANC* Ante-natal care, *Ref* Reference the logistic regression estimates are controlled for all the background characteristics

Women with age at first birth 25 years and above had 2.75 times significantly higher odds of delivering through elective c-section than women at first birth below 18 years [AOR: 2.75; CI: 1.67 -4.55]. However, the results were the opposite in the case of women who delivered through emergency c-sections. Children from 2^nd^ [AOR: 2.58; CI:1.94–3.43] and third or higher [AOR: 2.67; CI: 1.75–4.05] birth order were significantly more likely to get delivered through elective c-section than children from first birth order. However, Children from second [AOR: 0.39; CI: 0.29 -0.54] third or higher [AOR: 0.29; CI: 0.18 -0.45] birth order were significantly less likely to get delivered through emergency c-section than children from first birth order. The odds of getting delivered through either elective or emergency c-sections were significantly higher among women delivering in private health facilities than in public health facilities. Women from the richest wealth quintile had significantly higher odds of delivering through elective c-section than women from the poorest wealth quintile [AOR: 1.64; CI: 1.04–2.6]. However, wealth quintile was not associated with women delivering through emergency c-sections. Women from Chittagong [AOR: 0.62; CI: 0.41 -0.93] had significantly lower odds of delivering through c-section than women from Barisal.

## Discussion

Evidence from low-and-middle-income countries has often found the coexistence of both overuse and underuse of caesarean section delivery [36]. As a result, many women are left deprived with the lack of accessibility while others undergo the procedure unnecessarily [37, 38]. Literature shows that Asian countries like Iran have seen a surge in c-section practices over three decades [39]. One of the South Asian countries, Bangladesh, is no exception. Using the latest nationally representative survey, this study showed that about 32.8% of women have undergone c-section deliveries. Further, we explored the predictors associated with the timing of the c-section decision (i.e., elective and emergency c-section deliveries) among reproductive age-group women in Bangladesh.

Emergency and elective c-section deliveries are two surgical procedures performed to reduce the complications associated with childbirth. The present study found that more than 18% of Bangladeshi mothers underwent an elective c-section compared to 14.1% of mothers who underwent an emergency c-section. On the one hand, factors like the mother’s higher—age at birth and birth order increased the chance of elective c-section deliveries. Reverse results were found in case of emergency c-section deliveries. Elective and emergency c-section deliveries were higher among women who went to a private health facility for delivery.

The present study further found that educated women were lesser likely to undergo emergency c-section. A possible explanation could be that higher education among women can bring confidence and the capability to take action regarding their health [39]. Education can also bring the privilege of understanding the pros and cons of c-section deliveries among women. In contrast to the existing studies, our study found that the c-section delivery rate was higher among unemployed women [40–42]. This may be possible if women’s husbands have time constraints due to their working profession and, thus, prefer to have a c-section delivery. The present study also found a lower prevalence of c-section delivery among women who had unemployed husbands than those who were professionally employed. However, further investigation is required to better understand this finding by considering the context-specific factors.

Exposure to mass media plays an important role in choosing methods of delivery. As documented, women usually demand elective c-section when they find this procedure convenient, less painful, and easier, without understanding the complications attached to unnecessary procedures [43]. Our study consistently found that elective c-section was common among women who had exposure to mass media; however, emergency c-section delivery had no significant association. Being overweight is also a risk factor for c-section deliveries especially elective c-sections. This may be attributed to previous evidence that overweight or obese women have a higher risk of gestational diabetes and preeclampsia during pregnancy, leading to stillbirth and congenital anomalies [44]. The prior knowledge of such complications may contribute to opting for elective c-sections among women, as found in the past study [45]. Another contributing risk factor is women’s age during childbirth. The knowledge of pregnancy complications and fear of labor pain at higher ages usually make them opt for elective c-sections. The findings were consistent with a study in Bangladesh, where c-section deliveries were common among women of late reproductive age [25, 32]. The present study suggests that the odds of elective c-section deliveries increased with the increasing birth order of children. A possible explanation is that subsequent deliveries are presumed to have higher risks after the birth of the first or second child with a c-section. Also, the procedure’s efficacy to avert the risk from mother and child creates a positive perception of women towards this procedure, resulting in opting for c-sections for subsequent deliveries. Surprisingly, the chance of emergency c-sections in children with higher birth order decreases as women’s experience from their first child may provide knowledge for further deliveries [32].

With a dramatic spread of private facilities in Bangladesh, institutional deliveries have also increased in the last few decades [46]. The perception of improved quality of care, availability of specialist physicians, and lack of facilities in public health services have constantly driven the population to avail of private health facilities [47, 48]. Consistent with the prior studies in Bangladesh, the present study found a significant positive association between availing of private health facilities and opting for elective and emergency c-section deliveries [25, 32]. A possible explanation is the profit-driven nature of private facilities, highly triggered by the public initiatives for increased institutional delivery [49]. Although the institutional deliveries were first promoted to have safe childbirth, private facilities have used this to enforce higher c-section deliveries to save time and make money. Consistent with the prior studies, this study found that wealthy mothers have a higher chance of elective c-Sects. [32, 39]. Most of the administrative divisions of Bangladesh have shown a lesser chance of elective c-section deliveries. Several studies have shown that living in urban areas is highly associated with c-section deliveries [50, 51]. However, consistent with the present study, recent evidence from Bangladesh has shown that residing in urban areas has no association with c-Sects. [52]. The emerging health literacy about the procedures of c-section deliveries in Bangladesh might explain this. Studies from Bangladesh have shown that, over the last decade, female education and medical facilities have improved even in small towns and villages [31, 53]. The maternal and child health initiative has led to a tremendous decrease in the death of mothers and children. However, with the improvised health facilities, an increasing trend of unnecessary c-section deliveries has also spread all over Bangladesh. This study found that a large proportion of Bangladeshi mothers undergo c-section deliveries.

So far, many studies have reported the risk factors of c-section delivery in Bangladesh [1, 25, 32, 53]. But most of these studies did not estimate the predictors of the two c-section procedures (i.e., emergency and elective) separately, which is based on the timing of the c-section decision. This study comprehensively examined the socio-demographic and economic factors that may affect emergency and elective c-section deliveries differently by considering many confounding variables. Moreover, this study uses the most recent Bangladesh demographic and health survey to show the determinants of both c-section procedures.

However, regardless of these strengths, the study has limitations too. The available data did not allow us to examine all the aspects of pregnancy and delivery practices. The role of quality and cost of delivery services, cultural factors, and prejudices were also not included due to a lack of information in the survey. Also, the cross-sectional nature of the data does not allow us to draw any causal inferences. Future research may consider these and also the economic prospects of c-section deliveries, including the type of insurance and payment for c-sections. The higher proportion of elective c-sections in Bangladesh found in this study has raised the concern of deciding c-section way before the indication of complications. Such figures may also be affected by the factors like woman’s willingness, physician advice due to complications, and sometimes physicians’ motive of time-saving and making more money simultaneously [54]. Further, qualitative studies are required to understand better the reasons for higher c-section rates among women belonging to the higher socioeconomic group.

## Conclusion

Although c-section delivery emerged as a life-saving intervention, the overuse of such practice has created lucrative risks for the mother and unborn child. Our study suggests that women who are overweight, wealthy, and exposed to mass media have significantly higher rates of elective c-sections. The two c-section procedures were common among private health facilities, whereas higher education among women was negatively associated with emergency c-section deliveries. The study suggests that these factors might be considered for reducing the unnecessary c-section deliveries in Bangladeshi mothers. Besides, routine clinical audits are required in facilities to monitor the change in c-section deliveries. Proper sensitization of mothers and families should be conducted to enhance the knowledge of the unsafe nature of unnecessary c-section deliveries. Authorizations in case of over-use of elective and emergency c-sections should be observed to minimize the unnecessary c-sections and related complications and to increase normal institutional deliveries in Bangladesh.

## Data Availability

The study uses a secondary source of data that is freely available in the public domain through: https://dhsprogram.com/data/dataset/Bangladesh_Standard-DHS_2017.cfm?flag=0

## Abbreviations

C-section: Caesarean section
BDHS: Bangladesh Demographic Health Survey
NIPORT: National Institute for Population Research and Training
MoHFW: Ministry of Health and Family Welfare
ANC: Antenatal Care
AOR: Adjusted Odds Ratio
